# Extracellular DNA and Markers of Neutrophil Extracellular Traps in Saliva from Patients with Periodontitis—A Case–Control Study

**DOI:** 10.3390/jcm13020468

**Published:** 2024-01-15

**Authors:** Alexandra Gaál Kovalčíková, Bohuslav Novák, Oksana Roshko, Eva Kovaľová, Michal Pastorek, Barbora Vlková, Peter Celec

**Affiliations:** 1 Department of Pediatrics, National Institute of Children’s Diseases and Faculty of Medicine, Comenius University in Bratislava, 83340 Bratislava, Slovakia; alexandra.kovalcikova114@gmail.com; 2 Department of Stomatology and Maxillofacial Surgery, Faculty of Medicine, Comenius University, 81250 Bratislava, Slovakia; bohuslav.novak@fmed.uniba.sk; 3 Department of Dental Hygiene, Faculty of Health Care, Prešov University, 08001 Prešov, Slovakia; oksana.roshko@unipo.sk (O.R.); kovalova@nextra.sk (E.K.); 4 Institute of Molecular Biomedicine, Faculty of Medicine, Comenius University, 81108 Bratislava, Slovakia; michal.pastorek@imbm.sk (M.P.); barbora.vlkova@imbm.sk (B.V.); 5 Institute of Pathophysiology, Faculty of Medicine, Comenius University, 81108 Bratislava, Slovakia

**Keywords:** inflammation, alarmins, cell-free nucleic acids, biomarkers, immunogenic cell death

## Abstract

Periodontitis is a chronic inflammatory disease. We have previously shown that salivary DNA is higher in patients with periodontitis. Neutrophil extracellular traps (NETs) are involved in the pathogenesis of chronic inflammatory diseases. The objective of this case–control study was to compare patients with periodontitis and healthy controls regarding the salivary concentrations of extracellular DNA and NET components. Unstimulated saliva samples were collected from 49 patients with periodontitis and 71 controls before an oral examination. Salivary extracellular DNA was isolated and quantified fluorometrically and using PCR. NET-associated markers were assessed using ELISA. We have found significantly higher concentrations of salivary extracellular DNA in samples from periodontitis patients (five-times higher for supernatant and three times for pellet). Our results show that patients also have three-times-higher salivary nucleosomes and NET-associated enzymes—myeloperoxidase and neutrophil elastase (both two-times higher). Neutrophil elastase and salivary DNA in the pellet correlated positively with the pocket depth/clinical attachment level in periodontitis patients (r = 0.31—weak correlation; *p* = 0.03 and r = 0.41—moderate correlation, *p* = 0.004). Correlations between salivary extracellular DNA and NET enzymes were positive and significant. Based on our results, the higher salivary extracellular DNA in periodontitis seems to be related to components of NETs, albeit with weak to moderate correlations indicating that NETs are produced in periodontitis and can play a role in its pathogenesis similarly to other inflammatory diseases. Further studies should prove this assumption with potential diagnostic and therapeutic consequences.

## 1. Introduction

Despite decades of research, the etiopathogenesis of periodontitis is incomplete. The consequence is the lack of a specific causal treatment, and so scaling and root planing are still used as the primary approach [1]. Periodontitis is a chronic inflammatory disease, and despite the undoubted role of periodontal microbial pathogens, it can potentially share some of the pathomechanisms that are involved in inflammatory diseases affecting other joints or tissues [2]. Neutrophils play an important role in innate immunity and in chronic inflammation, including periodontitis [3]. The neutrophilic inflammation that potentially starts as a response to pathogens is successfully targeted in several diseases [4], and it is likely that similar processes are ongoing in the periodontium. Proving this in a clinical study can pave the way for new therapeutic targets and interventions.

Neutrophils have several very different weapons to control infections. Neutrophil extracellular traps (NETs) have been described as an antimicrobial mechanism used in the fight against microorganisms [5]. However, it has become clear that NETs are also involved in the vicious cycle of sterile inflammation [6]. Especially, if not degraded completely and quickly, NETs and NET components are recognized by Toll-like receptors on immune cells. It is not completely clear which components are relevant, but NETs are not only produced as a consequence of inflammation, but are also able to induce inflammation, including NET formations [7]. This vicious cycle seems to be involved in the pathogenesis of various chronic inflammatory diseases [8] and can be partly responsible for the inflammation of the periodontium as well [9].

NETs are web-like structures composed of extracellular DNA, partially in the form of nucleosomes, but also neutrophil enzymes, including myeloperoxidase and neutrophil elastase [10,11]. These can be analyzed with immunohistochemistry and are observed in biopsies from periodontitis patients [12]. A common way to quantify NETs is the measurement of extracellular DNA. Whether any of these components are important for the pathogenesis of inflammatory diseases is not known. The most important proof of the role of NETs comes from a recent experimental study indicating that mice not able to produce NETs or mice treated to better cleave NETs have reduced symptoms of ligation-induced periodontitis [13]. The clinical part of their study analyzed NET-associated biomarkers in serum and gingival crevicular fluid, but not in saliva.

Saliva is a noninvasive diagnostic fluid that can easily be collected, even at home by patients, without the need for trained personnel. This is of importance for screening, but also for monitoring disease progression [14]. We and others have shown that salivary DNA is higher in periodontitis [15,16,17]. This increased DNA can originate from the necrosis or apoptosis of damaged mucosal tissue, but also from activated immune cells undergoing NETosis or NETs production [18]. This is not yet clear, based on the published literature on salivary extracellular DNA. Inside the cell, the DNA is protected in the nucleus and in the mitochondria. These types of DNA can be released by different mechanisms and can induce different immune responses [19]. It is, thus, important to quantify the subtypes of DNA in addition to the total extracellular DNA in saliva.

In plasma, the preparation of cell-free extracellular DNA requires a two-centrifugation protocol to remove cells and cell debris. The centrifugation force and proper handling of the supernatant after centrifugation affect the quality and quantity of the analyzed nucleic acids [20]. It has been shown that at least a part of the DNA is in extracellular vesicles that protect the DNA from degradation [21]. On the other hand, it is not completely clear what is in the pellet after the second spin. Whether these are large vesicles or organelles from damaged cells is not clear [22]. However, the further specificity contributed to by the analysis of the vesicles in addition to the DNA itself can be very helpful in the analysis of fetal or tumor DNA.

The aim of this case–control study is to compare patients with periodontitis and healthy controls regarding the salivary concentrations of extracellular DNA and NET components, including nucleosomes, neutrophil elastase, and myeloperoxidase. We hypothesize that salivary extracellular DNA in periodontitis originates from NETs and that periodontitis is associated with increased salivary extracellular DNA as well as the analyzed NET components.

## 2. Materials and Methods

### 2.1. Patients

This is a report from an observational single-center case–control study. Unstimulated saliva samples were collected at a dental clinic from consecutive patients with periodontitis (*n* = 49) and from healthy controls recruited from patients, healthcare personnel, and family members (*n* = 71). The diagnostic procedure leaned on the actual diagnostic criteria for chronic periodontitis [1] and were conducted by one examiner (OR) under the guidance of senior experts in periodontology (EK, BN). The clinical recruitment and examination took place at a single center—the Department of Dental Hygiene at the Prešov University in 2018–2019. The basic characteristics of the participants are shown in Table 1. The patients were instructed to abstain from eating and drinking at least one hour before sampling. Saliva was collected without any external stimulation by drooling and spitting into sterile tubes. The samples were processed immediately. Routine periodontal examinations, including papillary bleeding index (PBI), clinical attachment level (CAL), and probing pocket depth (PD), were conducted after saliva collection as already described [23]. The inclusion criteria included PD > 3 mm on at least 4 teeth with a BOP and CAL of 3 mm. The exclusion criteria were systemic diseases requiring pharmacological or surgical treatments or any other oral pathology. The study was approved by the ethics committee of the Institute of Molecular Biomedicine, Comenius University on 7 March 2018 (Registration number 2018-3-1). All participants signed an informed consent form.

### 2.2. Salivary DNA

Fresh saliva was centrifuged at 1600× *g* for 10 min at 4 °C to spin down the cells. The supernatant was transferred into clean tubes, frozen, and transported into the laboratory. Thawed samples were used for another centrifugation at 16,000× *g* for 10 min at 4 °C to spin down the cell debris. From this processing step, both the supernatant and the pellet were used for DNA isolation with the protocol from the manufacturer (QIAmp DNA Mini kit, Qiagen, Hilden, Germany). The quantification of extracellular DNA was conducted using a fluorometric approach (Qubit high sensitivity dsDNA assay, Thermo Fisher Scientific, Waltham, MA, USA) and using quantitative PCR targeting unique nuclear and mitochondrial sequences as previously described [24]. PCR efficiency was between 90% and 110% based on the analyses of the standard dilutions. A melting curve analysis was conducted with the PCR products to confirm the specificity of the reaction. The coefficients of variation for the technical variability were 3% for fluorometry and 10% for PCR; the concentrations were expressed as ng/mL or genome equivalents (GE)/mL, respectively.

### 2.3. ELISA Assays

Nucleosomes were quantified using the cell death-detection ELISA kit (Roche, Basel, Switzerland). Arbitrary units (AUs) were used for the quantification since no consensus quantitative standard was available. The kit was based on the sandwich assay using antibodies against DNA and histones. Myeloperoxidase and neutrophil elastase were assessed using the corresponding DuoSet ELISA kits (R&D Systems, Minneapolis, MN, USA). Intra-assay and inter-assay coefficients of technical variation were below 5% and 10%, respectively.

### 2.4. Statistical Analysis

A comparison between the groups was conducted using a Student’s *t*-test. The correlation analysis was performed using a Pearson correlation test. This was enabled by the lack of significant differences between the observed and normal distributions as tested using the goodness-of-fit test for normality. *p*-values less than 5% were considered significant. The results are presented as the mean ± standard deviation. A statistical analysis was conducted using XLStatistics 5.71 (Rodney Carr, XLent Works, Deakin University, Warrnambool, Victoria, Australia). A multivariate analysis was conducted using the general liner model within SPSS version 21 (IBM, Armonk, NY, USA) taking into account age, sex, and the groups of patients.

## 3. Results

Patients with periodontitis and the healthy controls were not matched for age or gender, but were included in the study, consecutively explaining why there were significant differences in both factors. Healthy controls were younger and included more women (Table 1). PBI and PD were significantly higher in patients with periodontitis than in the healthy controls (*p* < 0.001). This was confirmed using a multivariate analysis with age and sex as covariates. Group (the classification into healthy controls and patients with periodontitis) was a highly significant factor affecting both the PBI (F = 17.4, eta = 0.33) and PD (F = 244.6, eta = 0.87).

**Figure 1:**
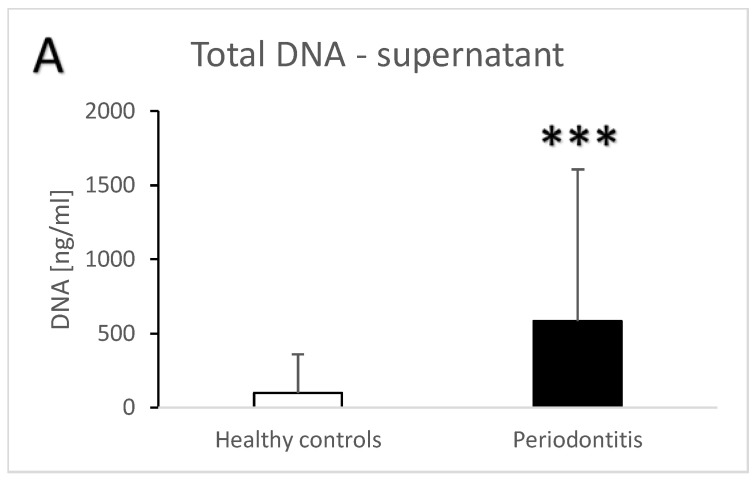

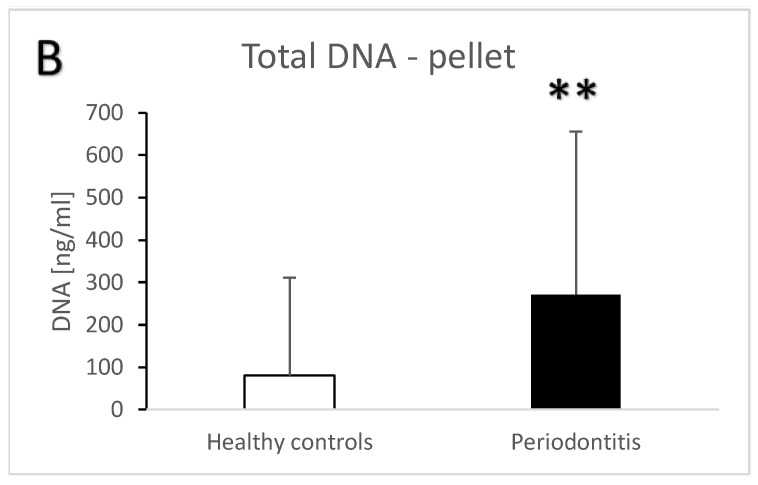
Total extracellular DNA in saliva supernatant (**A**) and pellet (**B**) samples from healthy controls and patients with periodontitis. **—*p* < 0.01, ***—*p* < 0.001.

**Figure 2:**
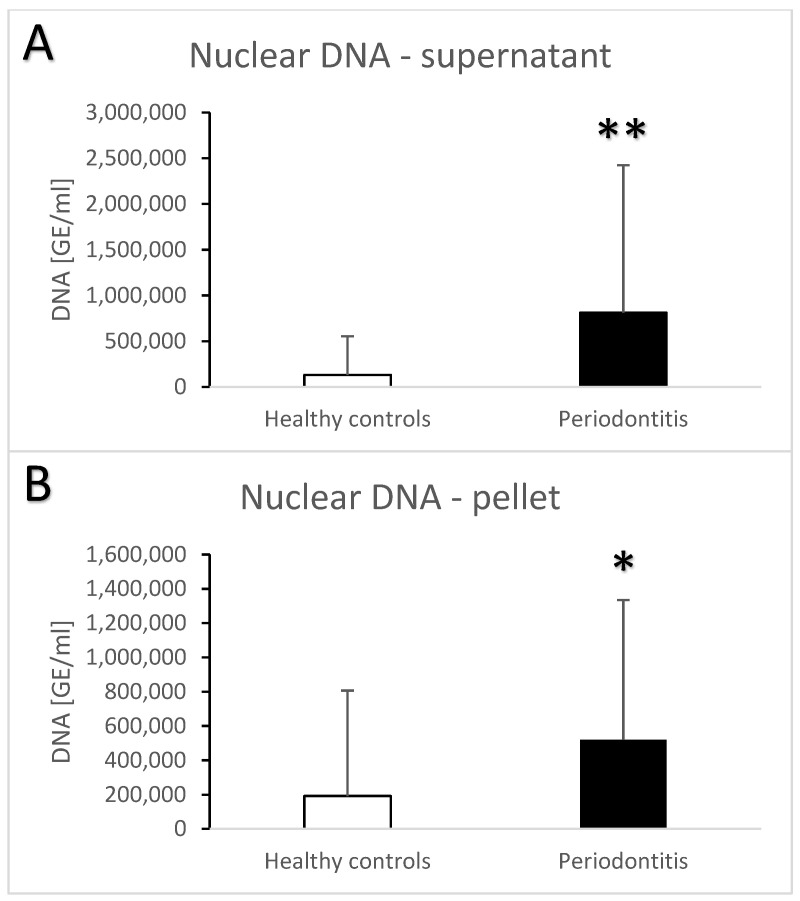
Nuclear extracellular DNA in saliva supernatant (**A**) and pellet (**B**) samples from healthy controls and patients with periodontitis. *—*p* < 0.05, **—*p* < 0.01.

**Figure 3:**
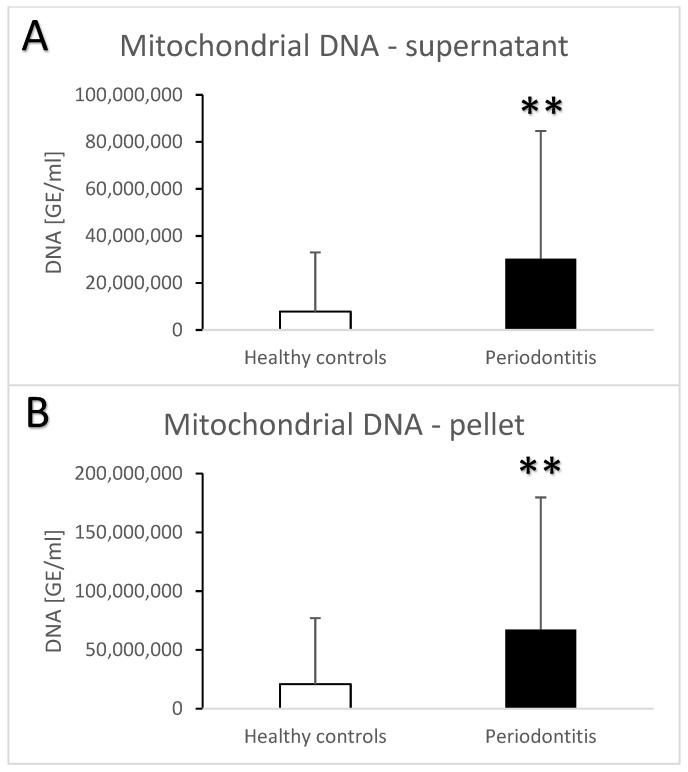
Mitochondrial extracellular DNA in saliva supernatant (**A**) and pellet (**B**) samples from healthy controls and patients with periodontitis. **—*p* < 0.01.

Periodontitis is associated with higher total salivary extracellular DNA (Figure 1A,B). Despite the high variability, the difference in comparison to the healthy controls was statistically significant with an average of 582 ± 1023 ng/mL vs. 100 ± 259 ng/mL (t = 3.7, *p* < 0.001) for the supernatant. A similar significant difference was found in the pellet—272 ± 384 ng/mL vs. 81 ± 231 ng/mL (t = 3.3, *p* < 0.01). Nuclear DNA quantified using real-time PCR revealed similar outcomes. In comparison to the healthy controls, patients with periodontitis had a higher copy number of nuclear DNA in the saliva supernatant (Figure 2A, t = 3.2, *p* < 0.01) as well as in the pellet (Figure 2B, t = 2.4, *p* < 0.05). While the interindividual variability for mitochondrial DNA was even higher than for nuclear DNA, the average copy number of mitochondrial DNA was more than three-times higher in patients with periodontitis than in healthy controls (Figure 3A, t = 3, *p* < 0.01 for supernatant, Figure 3B, t = 2.9, *p* < 0.01 for pellet). The differences between the groups were all confirmed, even when controlled for the age and sex of the patients. The relevant eta-squared coefficients for the group factor in all types of analyzed DNA were between 0.10 and 0.19 with *p*-values lower than 0.001.

**Figure 4:**
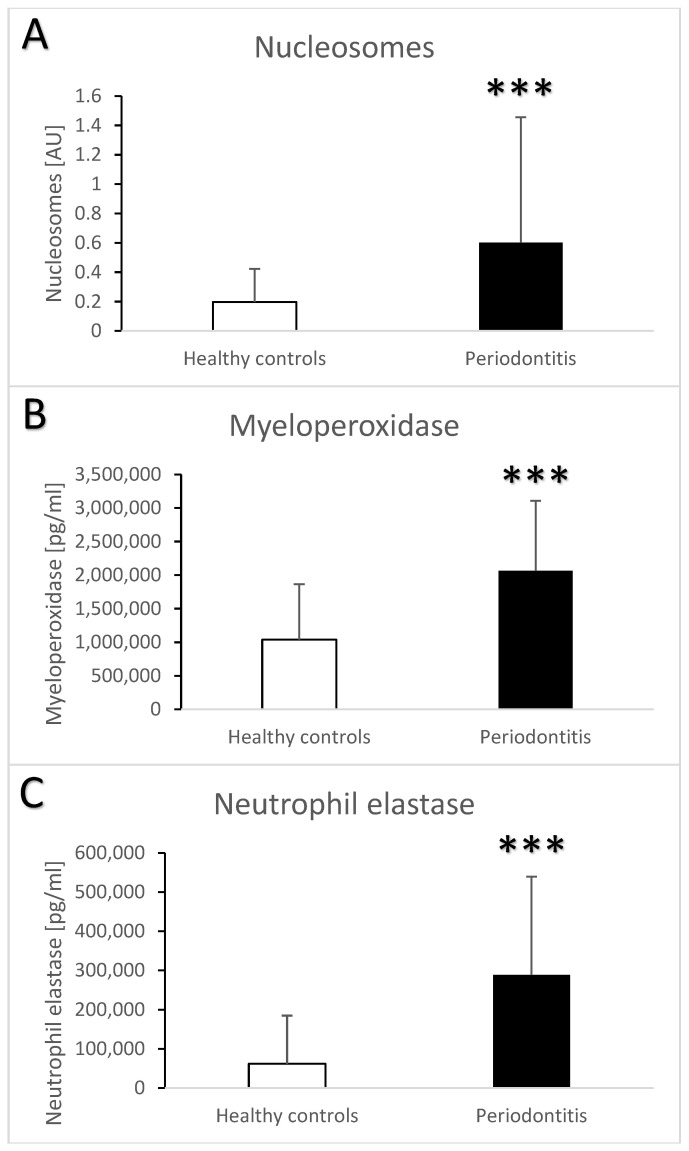
Salivary nucleosomes (**A**), myeloperoxidase (**B**), and neutrophil elastase (**C**) in saliva samples from healthy controls and patients with periodontitis. ***—*p* < 0.001.

The semi-quantitative ELISA for nucleosomes showed that the saliva of patients with periodontitis contained more nucleosomes than the saliva from the healthy controls (Figure 4A, t = 3.7, *p* < 0.001). The quantitation was limited by the lack of a quantitative standard, but the final concentration was likely three-times higher in the periodontitis vs. healthy controls. Myeloperoxidase and neutrophil elastase as neutrophil enzymes were also higher in the saliva from patients vs. controls. On average, both enzymes were two-fold more abundant in the saliva from patients with periodontitis (Figure 4B, t = 5.9, *p* < 0.001 for myeloperoxidase; Figure 4C, t = 6.4, *p* < 0.001 for neutrophil elastase. When controlled for the age and sex of the participants, the differences between the groups remained the major significant contributor to the variability for nucleosomes (F = 8, eta = 0.07, *p* < 0.01), myeloperoxidase (F = 15.5, eta = 0.0.13, *p* < 0.001), and neutrophil elastase (F = 19.1, eta = 0.15, *p* < 0.001). Among all the measured biochemical parameters, age significantly affected only neutrophil elastase based on the multivariate analysis (F = 6.2, eta = 0.05, *p* < 0.05). The correlation between age and neutrophil elastase in the saliva was positive, weak, and significant (r = 0.33, *p* < 0.05).

The correlation matrix of the clinical and salivary parameters analyzed in this study in samples from patients with periodontitis is shown in Table 2. A moderate correlation can be observed between PD/CAL and total salivary DNA in the pellet (r = 0.41, *p* < 0.01). None of the NETs markers or DNA correlate with the PBI, except for a weak negative correlation with nucleosomes (r = −0.32, *p* < 0.03). Moderate positive correlations exist between salivary DNA and NET-associated markers (Table 3). Total DNA in the supernatant weakly positively correlates with myeloperoxidase (r = 0.37, *p* < 0.001) and neutrophil elastase (r = 0.38, *p* < 0.001). Similarly, the DNA in the pellet is moderately associated with the neutrophil enzymes (r = 0.48, *p* < 0.001 for myeloperoxidase and r = 0.47, *p* < 0.001 for neutrophil elastase). Regarding the subcellular origin of the DNA, nuclear DNA, similarly to total DNA, correlates weakly positively with myeloperoxidase (r = 0.35, *p* < 0.001 for the supernatant and r = 0.39, *p* < 0.001 for the pellet) and moderately with neutrophil elastase (r = 0.45, *p* < 0.001 for the supernatant and r = 0.55; *p* < 0.001 for the pellet). Mitochondrial DNA correlates moderately positively with myeloperoxidase (r = 0.42, *p* < 0.01 for the supernatant and r = 0.45; *p* < 0.001 for the pellet), but not with neutrophil elastase. The associations with nucleosomes were statistically not significant.

## 4. Discussion

Our results are in line with previous, published findings of higher salivary extracellular DNA in patients with periodontitis [15,16,23]. The outcomes of our case–control study were confirmed using a multivariate analysis controlling for age and sex as covariates. Despite the lack of ideal matching in this study, the differences in salivary DNA and other NET components between the groups were proved to be independent of these factors and remained highly significant. To better characterize the subcellular origin of the salivary extracellular DNA, PCR targeting nuclear and mitochondrial DNA was used. The outcome suggests that both nuclear and mitochondrial DNA contribute to the difference in total salivary DNA, although the majority of salivary extracellular DNA is of a nuclear origin and the quantity of copies is higher for mitochondrial DNA by orders of magnitude. The various types of DNA in saliva correlate with neutrophil enzymes. This is in line with the hypothesis that NETs are an important source of salivary extracellular DNA in periodontitis. Similarly, the findings of higher nucleosomes and neutrophil enzymes as components of NETs in saliva from patients with periodontitis further strengthen this hypothesis.

The extracellular or cell-free DNA in plasma is widely studied and there are already clear consensus protocols for the processing of blood and plasma samples [25,26]. No such widely accepted protocols exist for saliva and salivary extracellular DNA. We, thus, applied the EDTA blood plasma protocol for saliva. The extracellular DNA is usually isolated from the supernatant after the second centrifugation. We decided to also isolate the DNA from the pellet containing cell debris described by some as microparticle-associated DNA [27]. The direct comparison shows that the pellet DNA in periodontitis patients shows similar differences to the true cell-free or extracellular DNA in the supernatant. Further analyses of the microparticles are needed in the future to better characterize their origins, including tests analyzing the presence of NETs in this compartment. Whether they might play a direct role in the pathogenesis of periodontitis is not clear. This aspect of the study is novel and can be seen as preliminary, given the lack of any previous analyses of microparticle-associated DNA in saliva.

The findings of this study indicate that NETs are the likely source of higher salivary DNA in periodontitis. However, the proof is missing. An interventional study with the blocking of NET production in patients with periodontitis is needed. The recently published experiments using genetic models as well as pharmacological interventions are convincing [13], but are based on an animal model of ligature-induced periodontitis, which, despite being widely used, has a different pathogenesis than the human disease [28]. This does not have to be an issue for the evaluation of NETs and NET-associated extracellular DNA as biomarkers of periodontitis. Answering the research questions related to the etiopathogenesis of the disease with a model that does not recapitulate the relevant disease pathomechanisms is, however, cumbersome.

In the literature, there are several reviews published about the role of NETs in periodontitis [9,29,30]. At the same time, however, there are virtually no human studies on NETs in saliva. One exception could be the study that focused on rheumatoid arthritis associated with periodontitis [31]. However, looking into the methods, the authors rather measured the total salivary DNA, but presented the concentration as NETs. Other studies analyzed NETs, but in biopsies from mice or patients, and not in saliva [32,33]. Interestingly, some authors measured NETs in blood serum/plasma from patients with periodontitis and found that circulating NETs were higher in patients vs. controls suggesting the systemic effects of this presumably local oral disease [32,34,35]. On the contrary, in patients with rheumatoid arthritis, these complexes presented as serum NETs correlated positively with PD [35].

The current trend is to use a combined ELISA with antibodies against myeloperoxidase and DNA for the assessment of NETs [36]. However, there are several technical obstacles that likely lead to a low specificity of the assay, as previously described in detail [37]. In our study, the quantitative aspects of this assay, even if using in vitro-produced NETs, were very limited, and it seemed it was not a local laboratory issue. This can explain why this ELISA is still not commercially used, despite hundreds of laboratories around the world working on NETs’ production, degradation, and their role in pathogenesis. In addition, the lack of a correlation between salivary nucleosomes and DNA or the negative correlation with PBI require further investigations, as any attempt to explain this based on the present results is pure speculation.

Based on our results, it is likely that increased salivary extracellular DNA in periodontitis stems from NETs. It is, however, not clear what should be the source of the NETs. It has been shown that some periodontal pathogens induce the formation of NETs when interacting with neutrophils [38]. Other pathogenic bacteria induce the formation of reactive oxygen species by neutrophils instead of NETs [39]. This is not mutually exclusive. Our previous research focused on oxidative stress markers in saliva in periodontitis [40,41]. This could be the consequence of bacteria- or NET-induced inflammation.

This study had several important limitations. The groups were not matched and, thus, a comparison could be biased. Dental plaque or oral hygiene indices were not included in the analysis as the study focused on the periodontal status. The changing classification of periodontitis was not reflected as staging and grading was not conducted. The observational nature of our study did not allow us to draw a conclusion regarding the causality of the identified associations. The concentrations of markers in saliva were neither normalized to salivation, which was not assessed, nor to total proteins, which could be biased, as well as shown for other biomarkers [42]. This study should test the practical applicability of the markers, which, if used in clinics, should be reported as the original concentrations. On the other hand, this study seems to be the first to study NET markers in saliva and not in serum or gingival crevicular fluid. A direct comparison of the different diagnostic fluids is needed to evaluate the specific advantages. The easy and non-invasive nature of saliva sampling makes saliva the most likely fluid to be used in the practice. The observed effects were convincing and consistent. A novel aspect is also the inclusion of total, nuclear, and mitochondrial DNA, which have divergent immune effects [43].

In conclusion, our results confirm that salivary extracellular DNA is high in periodontitis, regardless of its subcellular origin. In conjunction with the observed weak to moderate correlations of other analyzed NET components with the periodontal status, it is likely that NETs play a role as being a source of salivary extracellular DNA in periodontitis. Whether this association is causative and whether these NETs are involved in the pathogenesis of this chronic inflammatory disease is not clear. Future studies should test NET-targeting drugs for the treatment or prevention of periodontitis.

## Figures and Tables

**Table 1:** Patient characteristics included in the study.

Groups	Healthy Controls	Periodontitis
Number of subjects (n)	71	49
Age (years)	31.6 ± 9.5	48.5 ± 9.4
Papillary bleeding index	17.4 ± 11.8	41.0 ± 22.3
Probing depth (mm)	0.6 ± 0.4	6.2 ± 1.6
Clinical attachment level	-	3.2 ± 1.6
Bleeding on probing	-	1.4 ± 0.8

**Table 2:** Correlation matrix between clinical and biochemical parameters. Bold correlations are statistically significant and weak or moderate. s—supernatant, p—pellet.

		Total ecDNA—s	Total ecDNA—p	Nc ecDNA—s	Nc ecDNA—p	Mt ecDNA—s	Mt ecDNA—p	Myeloperoxidase	Neutrophil Elastase	Nucleosomes
Age	r	0.02	0.02	0.09	0.12	−0.09	0.04	−0.06	**0.33**	−0.09
	*p*	0.91	0.92	0.53	0.43	0.55	0.77	0.69	0.02	0.53
PBI	r	−0.08	0.00	−0.12	−0.17	−0.02	0.04	0.10	0.07	**−0.32**
	*p*	0.58	0.98	0.40	0.24	0.92	0.78	0.48	0.61	0.03
Probing depth	r	0.26	**0.41**	0.24	0.28	0.28	0.28	0.26	**0.31**	−0.07
	*p*	0.08	0.00	0.10	0.06	0.05	0.05	0.07	0.03	0.64
Clinicalattachment level	r	0.25	**0.41**	0.24	0.28	**0.29**	**0.29**	0.28	**0.31**	−0.08
	*p*	0.08	0.00	0.11	0.06	0.05	0.05	0.06	0.03	0.61

**Table 3:** Correlation matrix between salivary DNA and other components of neutrophil extracellular traps. Bold correlations are statistically significant. s—supernatant, p—pellet.

		Total ecDNA—s	Total ecDNA—p	Nc ecDNA—s	Nc ecDNA—p	Mt ecDNA—s	Mt ecDNA—p
Myeloperoxidase	r	**0.38**	**0.47**	**0.35**	**0.39**	**0.42**	**0.45**
	*p*	0.01	0.00	0.02	0.01	0.00	0.00
Neutrophil elastase	r	**0.37**	**0.48**	**0.45**	**0.55**	0.17	0.25
	*p*	0.01	0.00	0.00	0.00	0.24	0.08
Nucleosomes	r	0.13	0.04	0.17	0.13	−0.04	−0.09
	*p*	0.36	0.80	0.25	0.38	0.77	0.55

## Data Availability

The data obtained from this study are available from the authors upon reasonable request.
